# Sonographic nystagmus: a case report of lateral semicircular canal benign paroxysmal positional vertigo

**DOI:** 10.1186/s13089-025-00457-x

**Published:** 2025-10-14

**Authors:** Yuki Munekata, Chisato Matsumoto, Kento Sakoda, Yuki Takahashi

**Affiliations:** 1 Department of Emergency Medicine, Sunagawa City Medical Centre, 1-1, N4W3, Sunagawa, Sunagawa, 073-0196 Hokkaido Japan; 2 Department of Otolaryngology, Sunagawa City Medical Centre, Sunagawa, Japan

**Keywords:** Ocular ultrasonography, Ophthalmic ultrasound, Nystagmus, Benign paroxysmal positional vertigo, Vertigo, Emergency medicine, Frenzel goggles

## Abstract

**Background:**

Nystagmus, a critical diagnostic sign of vertigo and dizziness, reflects disturbances in the vestibular system. The accurate characterisation of nystagmus aids in distinguishing between peripheral and central causes, such as benign paroxysmal positional vertigo (BPPV) and stroke, respectively. Traditional methods for assessing nystagmus involve visual inspection or the use of Frenzel goggles. However, in cases where patients are unable to keep their eyes open owing to severe vertigo, such an evaluation becomes challenging. To date, sonographic imaging has not been used as a diagnostic method for nystagmus. We present a case in which ocular ultrasonography was used to assess nystagmus and aid in the diagnosis of BPPV.

**Case presentation:**

A 56-year-old woman presented with recurrent vertigo exacerbated by positional changes. Clinical examination revealed no spontaneous nystagmus or neurological deficits. Because the patient was unable to keep her eyes open during positional testing, ocular ultrasonography was performed using a 9-MHz linear transducer. Imaging revealed direction-changing horizontal nystagmus, consistent with the apogeotropic variant of lateral semicircular canal BPPV. The diagnosis was subsequently confirmed by an otolaryngologist using Frenzel goggles. The patient was conservatively managed.

**Conclusions:**

This is the first report on the use of ocular ultrasonography to assess nystagmus. Despite certain limitations, this technique may be a valuable supplementary tool, particularly in settings in which conventional examinations are hindered or unavailable. Ophthalmic sonography has the potential to enhance vestibular assessments in emergency and outpatient settings.

**Supplementary Information:**

The online version contains supplementary material available at 10.1186/s13089-025-00457-x.

## Background

Nystagmus is characterised by ocular movements with alternating fast and slow components. These movements give the appearance that the eyes are “beating” in the direction of the fast phase. Nystagmus is most often caused by an imbalance in the peripheral or central vestibular system [1]. Assessing a patient for nystagmus plays a crucial role in determining the cause of dizziness because these movement patterns are hallmark features of various disorders [2, 3]; this can help identify patients who likely have a dangerous central cause, such as stroke. However, central lesions can closely mimic peripheral lesions [1, 4]. Benign paroxysmal positional vertigo (BPPV) is the most common type of peripheral vestibular vertigo [5–7].

Traditionally, nystagmus has been assessed using Frenzel goggles or, in some cases, by direct visual inspection with the naked eye in the emergency department or otolaryngology outpatient settings. However, several challenges are associated with the assessment of nystagmus. First, many patients experience difficulty keeping their eyes open during the assessment, particularly when experiencing nausea associated with dizziness or vertigo. If a patient is unable to open their eyes, evaluating their ocular movements in detail is not possible. Similarly, traditional methods for assessing nystagmus may be limited in individuals with severe ptosis or significantly narrow palpebral fissures. Second, Frenzel goggles may not always be available, particularly in the emergency department. Under such circumstances, clinicians must rely on direct visual observations with the naked eye, which makes it more challenging to accurately assess the quality and direction of ocular movement. To date, no published reports have described the use of sonographic imaging to evaluate nystagmus. In this case report, we present a novel approach in which ocular ultrasonography was used to assess nystagmus and aid in the diagnosis of BPPV.

## Case presentation

A 56-year-old woman was admitted to our institution with the primary complaint of vertigo. These episodes were provoked by positional changes, such as the transition from a supine to an upright position. Each episode of vertigo lasted approximately 30 seconds and was not accompanied by hearing loss or tinnitus. Her medical history included BPPV, which was diagnosed approximately 10 years prior.

On presentation, her vital signs were: Glasgow Coma Scale score of E4V5M6, respiratory rate of 10–20 breaths per minute, oxygen saturation of 98% on room air, heart rate of 101 beats per minute, blood pressure of 128/56 mmHg, and body temperature of 36.2 °C. A physical examination revealed no spontaneous nystagmus at rest. Head impulse test results were normal and no skew deviation was observed. Neurological examination results were unremarkable and no focal neurological deficits were identified. Her gait was stable and unremarkable.

During the supine roll test, the patient was unable to keep her eyes open because of severe vertigo. Therefore, ocular movements were assessed using a commercially available sonographic imaging device during the supine roll test and Dix–Hallpike manoeuvre. A 9-MHz linear transducer was used for the ultrasound examination, with a mechanical index (MI) of 0.23 and thermal index (TI) of 0.0. During the ultrasound examination, only a minimal amount of gel directly onto the closed eyelid, to avoid infiltration beneath the lid margin. Initial assessment confirmed the absence of nystagmus-like eye movements at rest (Additional file 1: Video S1; Video S2). Subsequently, the supine roll test elicited right-beating horizontal nystagmus when the patient’s head was turned to the left (Additional file 1: Videos S3, S4) and left-beating horizontal nystagmus when the head was turned to the right (Additional file 1: Video S5, Video S6). The Dix–Hallpike manoeuvre did not induce nystagmus or sickness.

Based on sonographically observed nystagmus, clinical history, and examination findings, a provisional diagnosis of apogeotropic lateral semicircular canal BPPV (LC-BPPV) was made. The patient was subsequently referred to the Department of Otolaryngology for further evaluation. An otolaryngologist reviewed the patient and confirmed the presence of direction-changing apogeotropic horizontal positional nystagmus during a supine roll test using Frenzel goggles. The observed vertigo was characterised by latency and attenuation. Consequently, the diagnosis of apogeotropic LC-BPPV was confirmed and the patient was managed conservatively.

## Discussion

In the present case, nystagmus was identified using ophthalmic sonography, which contributed to the provisional diagnosis of LC-BPPV. To the best of our knowledge, this is the first report describing the visualisation of nystagmus using sonographic imaging.

BPPV is caused by free-floating otoconia that have been dislodged from the otolith organs or, more rarely, otoconia that have adhered to the cupula [5–8]. Posterior canal BPPV is the most common, followed by LC-BPPV [7, 8]. LC-BPPV is characterised by a horizontal nystagmus elicited during a supine roll test. Geotropic nystagmus “beats” towards the ground, whereas apogeotropic nystagmus “beats” towards the ceiling [8]. Nystagmus assessment and interpretation are essential for the management of BPPV [1, 5]. In our case, a detailed assessment of nystagmus was achieved using ocular ultrasonography, even when the patient was unable to keep her eyes open during nystagmus-inducing manoeuvres, such as the supine roll test and Dix–Hallpike manoeuvre. This represents a major advantage of ultrasonographic examination. Furthermore, ultrasonography is a noninvasive and widely accessible modality, making it a practical tool in emergency and outpatient settings.

In recent years, ocular ultrasonography has been widely used because of its non-invasiveness, speed of execution, and quickly obtainable results; moreover, these results are useful in the diagnosis of different diseases, such as idiopathic intracranial hypertension through the assessment of the optic nerve sheath diameter [9, 10]. Lahham et al. reported that point-of-care ultrasonography performed by emergency medicine physicians may be a useful adjunct for the diagnosis of retinal detachment, vitreous haemorrhage, and vitreous detachment [11]. Several researchers have highlighted the invaluable utility of ultrasound in the assessment of occult intraocular foreign bodies [12, 13]. Additionally, pupillary light reflex may be effectively measured with ultrasound examination [14]. Although ultrasonography has been used in various ophthalmic assessments, its use in evaluating nystagmus has not been previously reported.

The key difference between Frenzel goggles and ultrasonography is that the former requires that patients keep their eyes open, while the latter can be performed with the eyelids closed. Both approaches present distinct benefits and drawbacks. Using the Frenzel goggles allows a detailed assessment of nystagmus in all planes, including torsional movements, though patients may experience discomfort in keeping their eyes open. Conversely, ocular ultrasonography can be conducted with closed eyelids, making it generally more comfortable. It also permits measurement of the optic nerve sheath diameter. This provides valuable information regarding elevated intracranial pressure, which is particularly relevant when evaluating possible central causes of nystagmus, such as stroke [15].

However, there are several important caveats associated with the use of ophthalmic sonography for nystagmus evaluation. The examination is typically conducted with a 7–15-MHz, small-footprint linear transducer coupled to a closed eyelid with a copious amount of gel to permit successful visualisation without excessive pressure on the globe [16]. The cornea and lens are more sensitive to sonographic exposure than the surrounding tissues. Ultrasound intensity varies with space and time. Sonography is considered safe within the diagnostic range. Therefore, there is strict adherence to the as low as reasonably achievable principle when performing ophthalmic sonographic procedures. According to the Food and Drug Administration guidelines on diagnostic ultrasound for ophthalmic applications, the following limits should be observed: the TI should not exceed 1, spatial peak temporal average intensity (ISPTA.3) should not exceed 50 mW/cm², and MI should not exceed 0.23 [16–18].

Another important caveat concerns the interpretation of nystagmus observed on ocular ultrasonography. Sonographic imaging provides a clearer visualisation of the posterior segment of the eyeball than the anterior segment. Consequently, the examiner’s focus is often directed towards the movement of the posterior aspect, which moves in the direction opposite to that used in conventional nystagmus assessment. For example, if the posterior part of the eyeball moves to the left during the fast phase on ultrasound, it should be interpreted as right-beating nystagmus, according to standard clinical definitions. Regarding factors contributing to the poor visualisation of the anterior chamber, the following were identified in our case. First, the amount of gel applied was deliberately reduced to prevent leakage, which may have been insufficient for optimal image quality. Second, excessive pressure may have been inadvertently applied to the globe because the examiner was simultaneously required to reposition the patient’s head during the examination. To improve visualisation, we suggest applying an adequate amount of gel along with an occlusive dressing to prevent leakage. In addition, a two-person technique, in which one examiner holds the probe while another repositions the patient’s head, could be considered. Finally, optimising machine settings, such as gain, may also enhance image quality.

Furthermore, conventional ophthalmic sonography is limited to two-dimensional assessments, typically capturing movements along the horizontal (left–right) or vertical (up–down) axes. As a result, vertical nystagmus may be overlooked if the probe is oriented horizontally relative to the palpebral fissure, although this limitation can be addressed by adjusting the orientation of the probe to align with the expected axis of ocular movement.

Finally, since this is the first case report on the sonographic evaluation of nystagmus, further case series and supporting evidence are needed to explore the precise value of ocular sonography in nystagmus assessment.

## Conclusion

Herein, we report the first known case of nystagmus assessed using ophthalmic sonography. Although there are inherent limitations and interpretative caveats associated with the use of ocular ultrasonography for nystagmus evaluation, this modality offers distinct advantages and has potential as a supplementary tool for the assessment and management of vertigo and dizziness accompanied by nystagmus. We consider ultrasonography to be particularly useful in situations where patients cannot keep their eyes open during assessment, especially when repeated manoeuvres elicit sustained nystagmus.

## Supplementary Information

Below is the link to the electronic supplementary material.

Supplementary Material 1. Additional File 1: Video S1. Patient’s right eye exhibited no characteristic movements at rest. In the ultrasound image, the left side of the screen corresponds to the patient’s right side. Video S2. Patient’s left eye exhibited no characteristic movements at rest. In the ultrasound image, the left side of the screen corresponds to the patient’s right side. Video S3. Right-beating horizontal nystagmus was observed in the patient’s right eye when the head was turned left. In the ultrasound image, the left side of the screen corresponds to the patient’s right side. Video S4. Right-beating horizontal nystagmus was observed in the patient’s left eye when the head was turned left. In the ultrasound image, the left side of the screen corresponds to the patient’s right side. Video S5. Left-beating horizontal nystagmus was observed in the patient’s right eye when the head was turned right. In the ultrasound image, the left side of the screen corresponds to the patient’s right side. Video S6. Left-beating horizontal nystagmus was observed in the patient’s left eye when the head was turned right. In the ultrasound image, the left side of the screen corresponds to the patient’s right side.

## Data Availability

The datasets used and/or analysed during the current study are available from the corresponding author on reasonable request.

## Abbreviations

BPPV: Benign paroxysmal positional vertigo
LC-BPPV: Lateral semicircular canal benign paroxysmal positional vertigo
TI: Thermal index
MI: Mechanical index
