# Supplementary material for: Sonographic nystagmus: a case report of lateral semicircular canal benign paroxysmal positional vertigo
Source: Ultrasound J. 2025 Oct 14;17:51. doi: 10.1186/s13089-025-00457-x (PMC12521668; doi:10.1186/s13089-025-00457-x)
Supplement: Supplementary file 1 — Supplementary Material 1. Additional File 1: Video S1. Patient’s right eye exhibited no characteristic movements at rest. In the ultrasound image, the left side of the screen corresponds to the patient’s right side. Video S2. Patient’s left eye exhibited no characteristic movements at rest. In the ultrasound image, the left side of the screen corresponds to the patient’s right side. Video S3. Right-beating horizontal nystagmus was observed in the patient’s right eye when the head was turned left. In the ultrasound image, the left side of the screen corresponds to the patient’s right side. Video S4. Right-beating horizontal nystagmus was observed in the patient’s left eye when the head was turned left. In the ultrasound image, the left side of the screen corresponds to the patient’s right side. Video S5. Left-beating horizontal nystagmus was observed in the patient’s right eye when the head was turned right. In the ultrasound image, the left side of the screen corresponds to the patient’s right side. Video S6. Left-beating horizontal nystagmus was observed in the patient’s left eye when the head was turned right. In the ultrasound image, the left side of the screen corresponds to the patient’s right side. [file 13089_2025_457_MOESM1_ESM.pptx]

## Slide 1
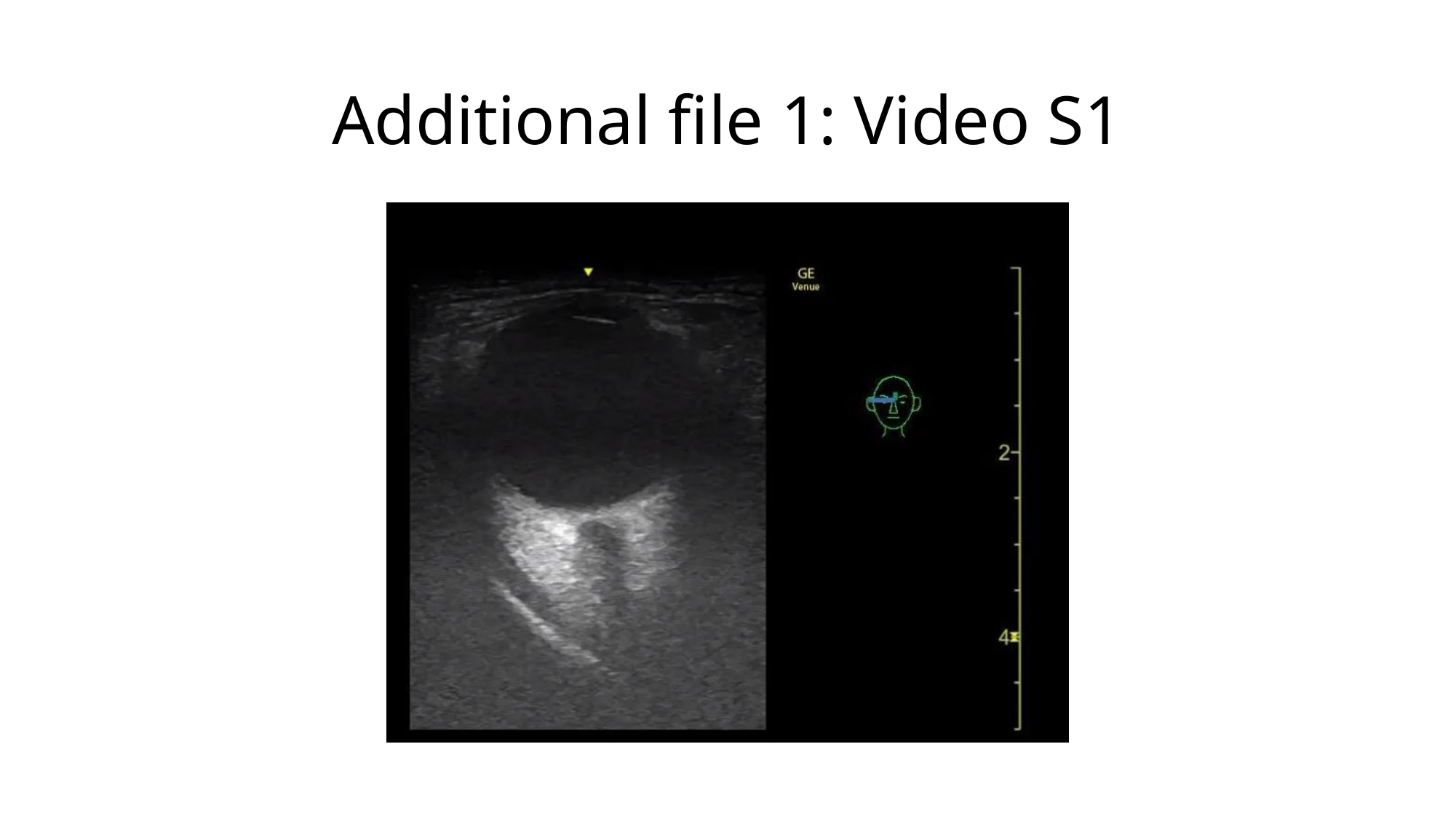

# Additional file 1: Video S1

## Slide 2
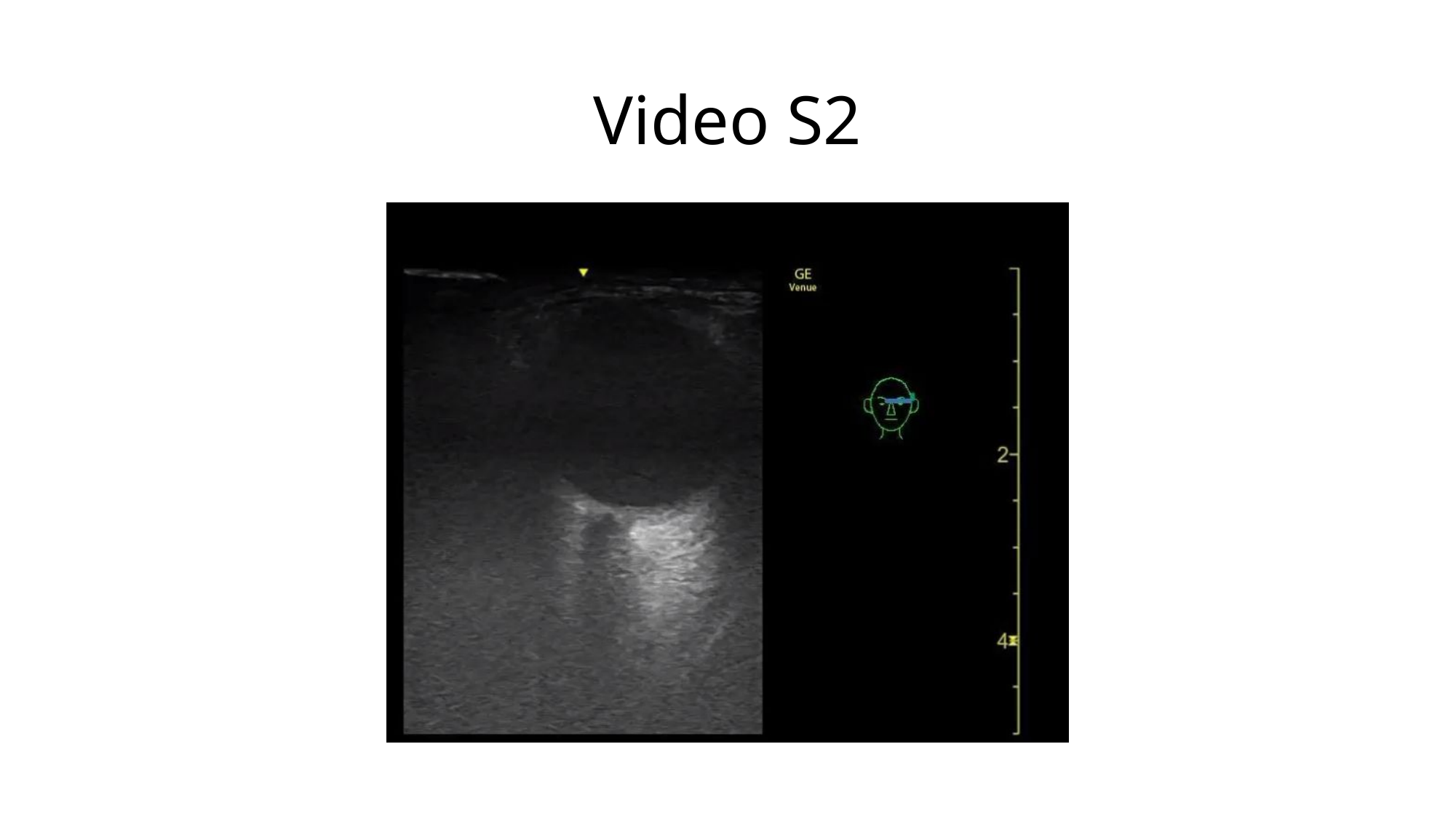

# Video S2

## Slide 3
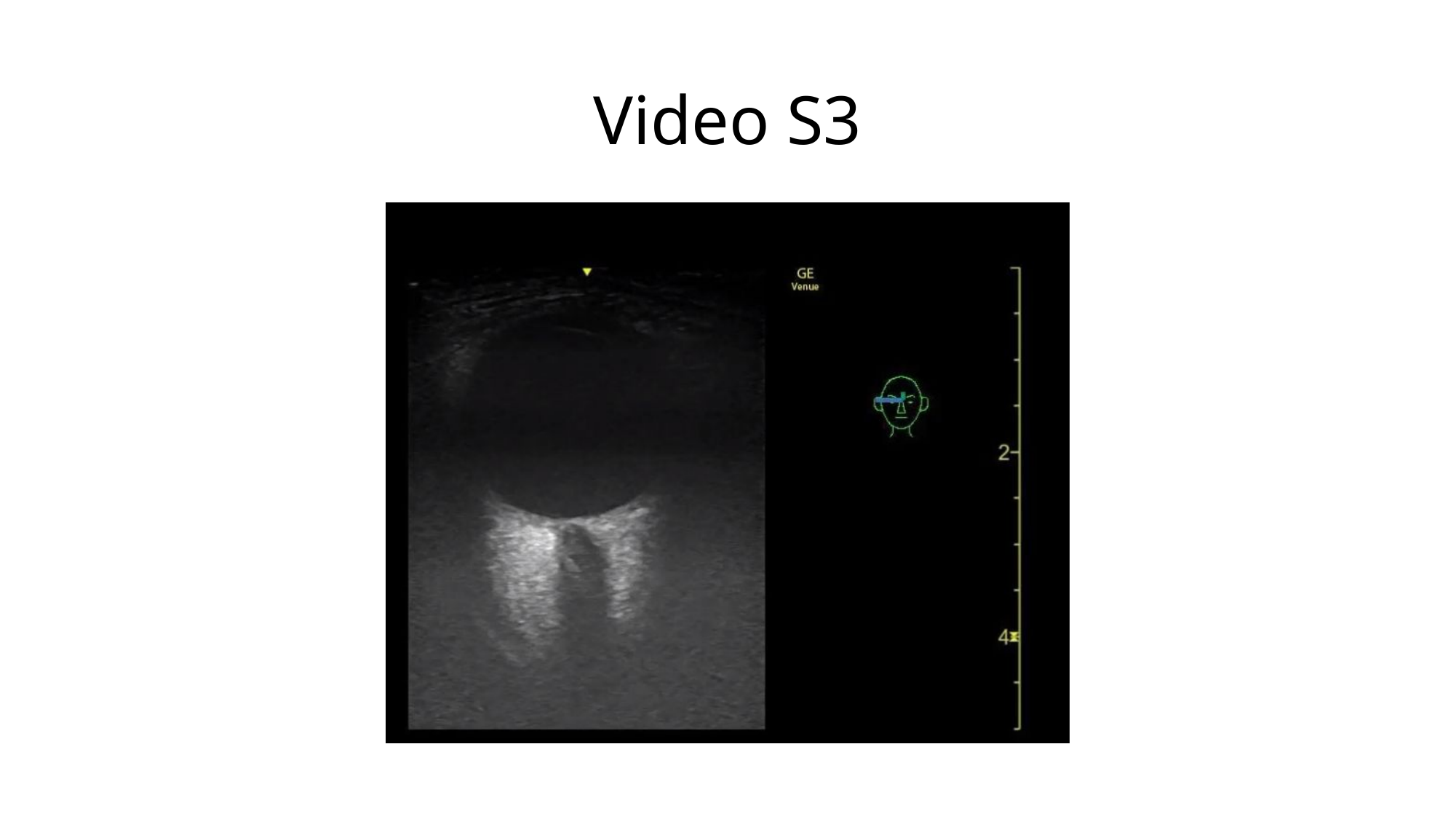

# Video S3

## Slide 4
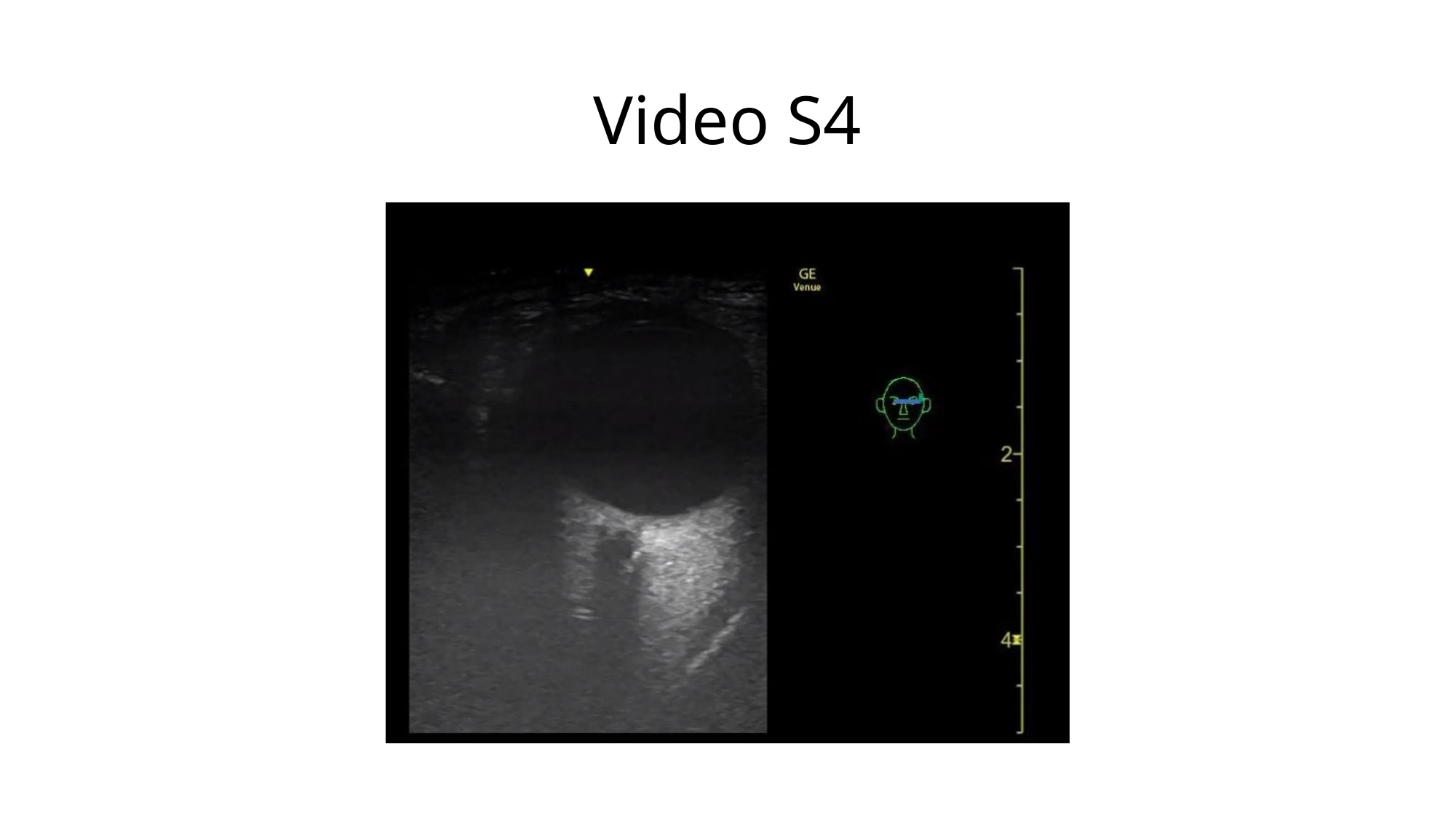

# Video S4

## Slide 5
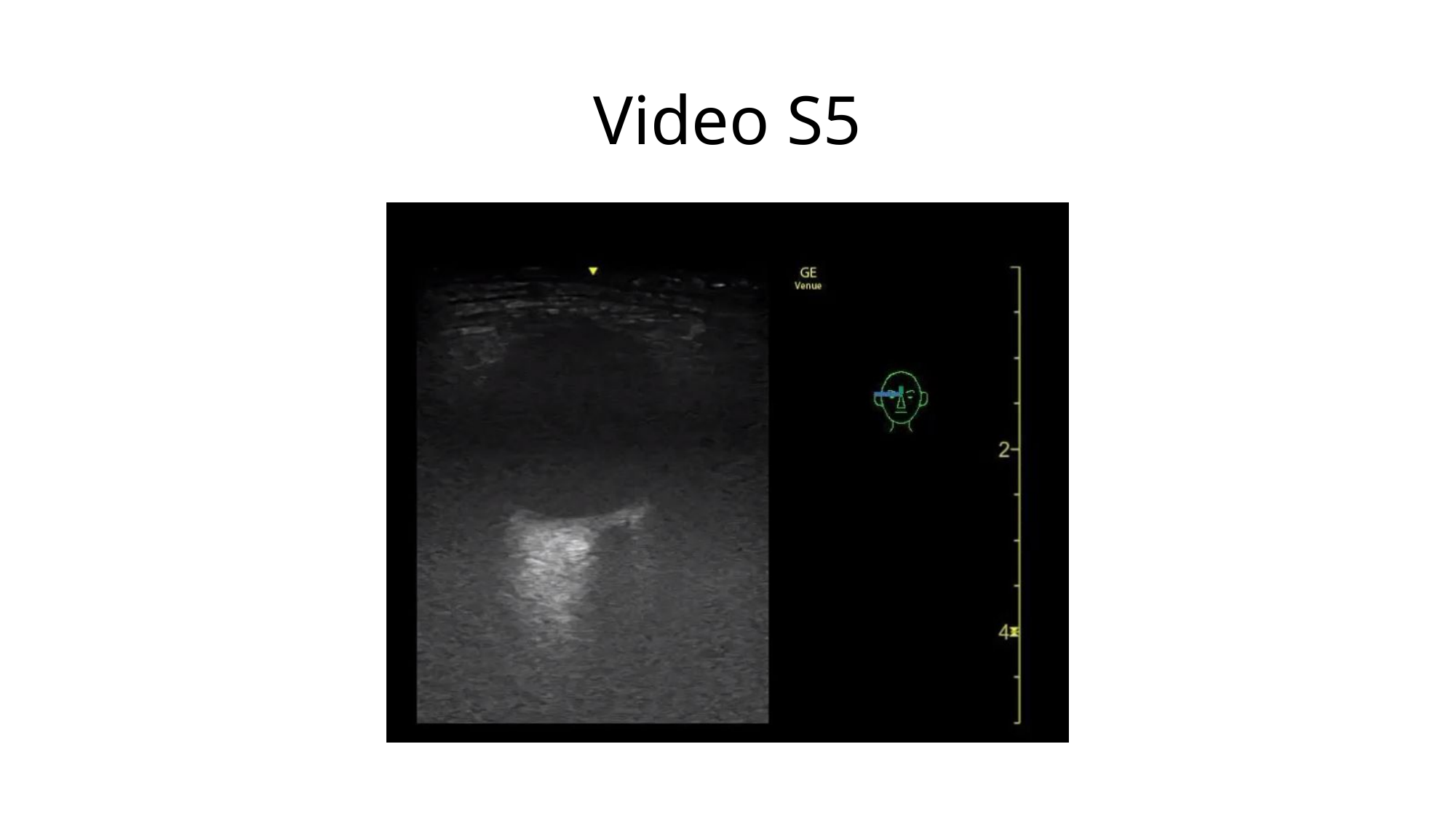

# Video S5

## Slide 6
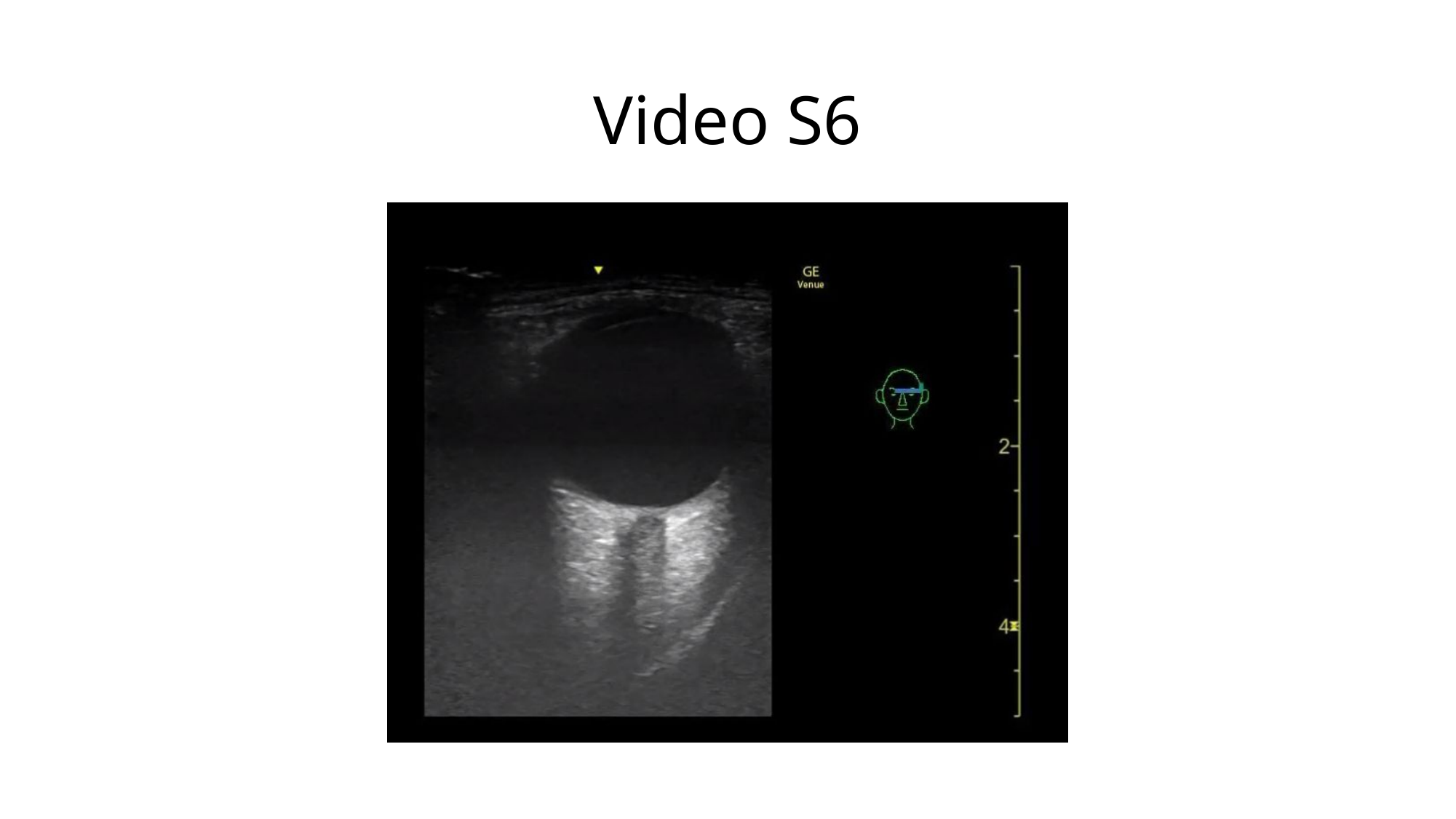

# Video S6
